# Decision-Making in Endodontics Prior to the Surgical Removal of Large Cysts Involving Vital Teeth

**DOI:** 10.7759/cureus.67665

**Published:** 2024-08-24

**Authors:** Khalid A Merdad, Khalid Al Johani, Maha Shawky, Haya AlSharif, Leen Mirdad, Mohsen Daghustani, Rahaf Alhabbab, Omar Alkhattab, Abdulaziz Bakhsh

**Affiliations:** 1 Department of Endodontics, King Abdulaziz University, Jeddah, SAU; 2 Department of Oral Diagnostic Sciences, King Abdulaziz University, Jeddah, SAU; 3 Department of Oral Maxillofacial Surgery, King Abdulaziz University, Jeddah, SAU; 4 Department of Dentistry, King Abdulaziz University, Jeddah, SAU; 5 Department of Endodontics, Jeddah Specialty Dental Centre, Ministry of Health, Jeddah, SAU; 6 Department of Oral and Maxillofacial Surgery, King Fahad General Hospital Jeddah, Ministry of Health, Jeddah, SAU; 7 Department of Restorative Dentistry, Umm Al-Qura University, Makkah, SAU

**Keywords:** decision-making, endodontics, root canal treatment, vital tooth, large cyst

## Abstract

Introduction

Maintaining pulp vitality is crucial; however, treatment options for large lesions may hinder the vitality of the teeth involved within the lesion. Some clinicians are not updated about the diagnostic terminology of the American Association of Endodontists (AAE) which may affect their decision-making. In the literature, there was no absolute treatment to manage such cases. The purpose of this study is to explore different opinions, identify the evidence of practice and treatment options to help in decision-making and assess if clinicians are acknowledged by the AAE guidelines.

Materials and methods

This cross-sectional study was conducted through a qualitative survey designed to interview randomly selected endodontists, oral maxillofacial surgeons, oral medicine specialists, oral pathologists, and general partitioners to record their decisions and management about a structured case scenario. A total of 120 participants were included in the study. The interviews were conducted by a single investigator, and the answers were recorded by another investigator. Finally, the responses of the interviewees were collected using Google Forms (Google, Mountain View, California).

Results

We found that there are dissimilarities between the different groups in decision-making concerning the management of teeth involved in large cystic lesions. Regarding the AEE guidelines, almost all the endodontics and general dentists were aware of the guidelines when compared with the other groups.

Conclusion

The management of teeth involved in large cystic lesions is controversial. Furthermore, the AEE guidelines are not a common language between the different disciplines. Randomized clinical trials are needed to investigate the prognosis and management of teeth associated with large cystic lesions.

## Introduction

Cystic lesions are pathological cavities filled with fluid, semi-fluid, or gaseous content that are not created by pus accumulation [1]. Most jaw cysts are lined with epithelium and expand by either hydrostatic and/or osmotic pressure.

Odontogenic cysts may cause issues, including facial deformity, occlusal disharmony, teeth deflection or displacement, and delayed eruption [2]. The treatment of cystic lesions depends on several factors, including the patient's age, root development, size and location of the cyst, and tooth vitality. Common treatment methods include extraction, coronectomy, root canal treatment (RCT), and revascularization, often coupled with surgical techniques such as enucleation and marsupialization [2].

Radicular cysts are known to be the most common odontogenic cysts, comprising 52% to 68% of jaw cysts [3]. Their incidence peaks in the third and fourth decades of life with male predominance. It originates from residues of epithelium in the periodontal ligament due to periapical periodontitis following pulp necrosis [3,4].

Periapical diseases are often associated with a necrotic pulp. Therefore, periapical lesions are usually treated with a conservative approach, including non-surgical root canal treatment [4,5]. However, management of inflammatory cysts involves root canal treatment for the necrotic tooth followed by surgical excavation of the non-regressing cyst [4-6].

In the literature, it is clear that non-vital teeth associated with inflammatory cysts require non-surgical and/or surgical root canal treatment. However, there is a lack of evidence in managing vital teeth associated with cystic lesions. In addition, clinicians must differentiate between the odontogenic and non-odontogenic cysts to determine the appropriate treatment approach. Therefore, the aim of this study is to explore the different opinions, experiences, routine practices, and treatment options of clinicians in the detection and management of teeth associated with cystic lesions.

## Materials and methods

Personal interviews were conducted with randomly selected groups of endodontists, oral maxillofacial specialists, oral medicine specialists, oral pathologists, and general dental practitioners, recording their clinical judgments, opinions, as well as their treatment plan based on their experience and exposures regarding the management of the teeth associated with a large cystic lesion.

Ethical approval

The protocol of this study was reviewed and accepted by the King Abdulaziz University Ethics Committee (no. 198-1-21).

Data collection

Data were collected using a self-designed questionnaire which is structured based on the main information of the endodontic diagnostic terminology of the American Association of Endodontists (AAE). A digital questionnaire was conducted using Google Forms (Google, Mountain View, California) to record all the information extracted from the interviewees. The questionnaire and the interview were in English. 

A pilot study was conducted and revised before distribution. Instrument revision included modifications to the questionnaire item's wording and format based on the recommendations to ensure feasibility, practicability, validity, and interpretation of the answers.

Sample size calculation

A Chi-squared sample size calculator (goodness of fit test) was used to determine the sample size of participants to be interviewed. Four categories of specialty with a maintaining significance level of p-value (0.05) resulted in 120 samples that will have a power equal to 0.8. 

Interviewing process

**Figure 1:**
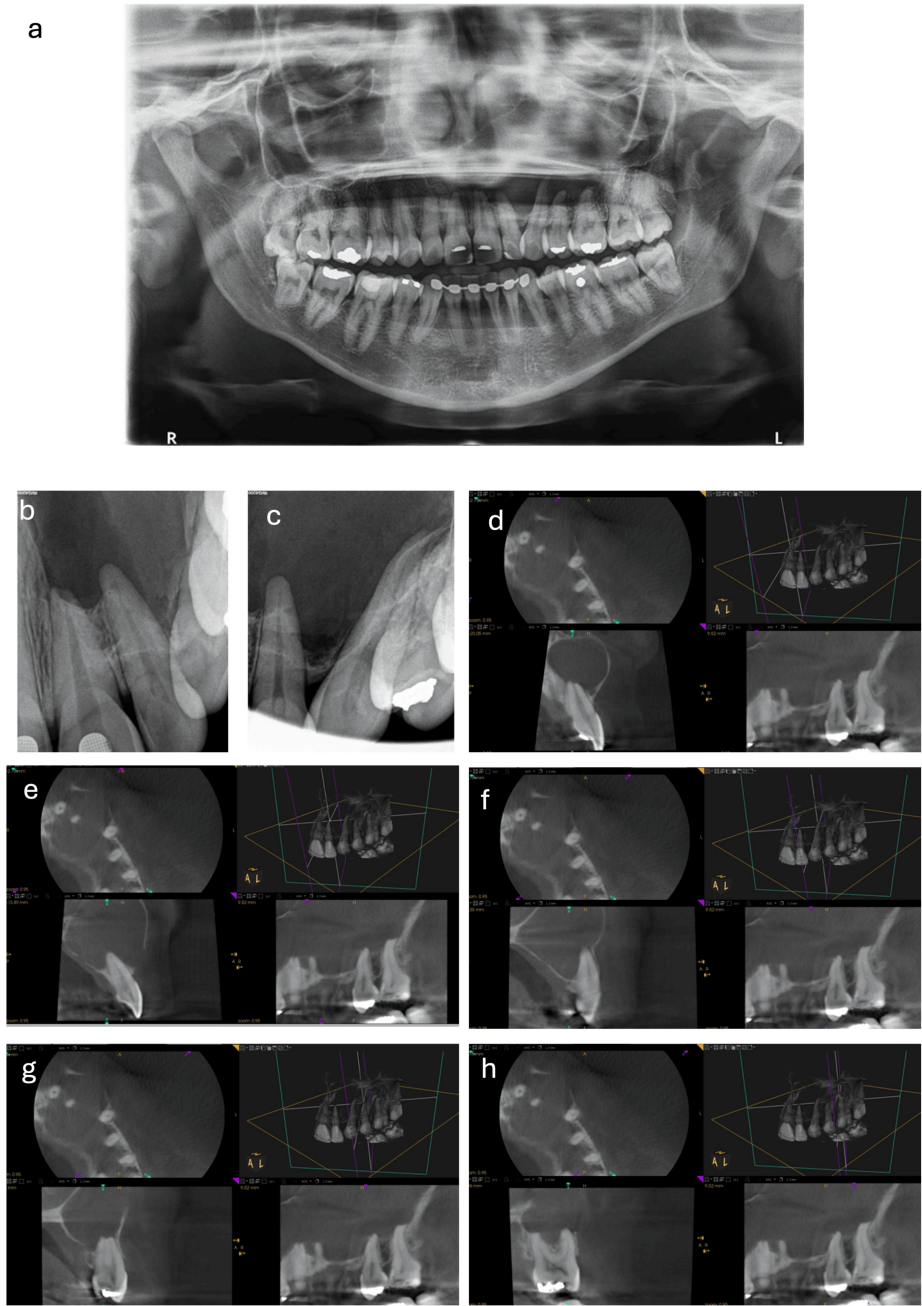
Case scenario A 35-year-old female patient (ASA I) came to the dental clinic complaining of pain and swelling. The pain, during biting, and the swelling are related to the upper left central maxillary incisor extending to the upper left first molar. Her medical & social histories are insignificant. She had a history of trauma. Clinical examination revealed: an eggshell crackling swelling covered with normal mucosa. Aspiration resulted in clear cystic fluid. Radiographic examination showed: A well-defined radiolucent lesion surrounded with a radiopaque line involving teeth from #21 to #26. Teeth from #23 to #26 are within normal limits (WNL). Tooth #21 responded negative to cold and electric pulp test (EPT) with mild pain on percussion. Tooth #22 responded with lingering pain to cold test and normal response to percussion. (a) Panoramic radiograph; (b,c) pre-operative periapical radiographs; (d) Cone beam computed tomography (CBCT) image of tooth #21; (e) CBCT of tooth #22; (f) CBCT of tooth #23; (g) CBCT of tooth #25; (h) CBCT of tooth #26.

All participants were consented to obtain approval to participate in the study. The face-to-face interview was carried out on an individualized basis, and interviewees were not allowed to consult any source of information at the time of the study. A single investigator was assigned to take over the interviews, and another one to record the answers from the interviewees, hence standardizing the variables and minimizing any potential impact of individual interviewers' behavior on respondents' answers and the resulting data. The interview consisted of four sections, including consent, demographic data of the participant, an original case scenario (Table 1, Figure 1), and finally, 14 self-explanatory questions about managing the presented case. Each interview was approximately 10-15 minutes long and was in English.

**Table 1:** Clinical responses to endodontic diagnostic tests EPT - electric pulp test; N - normal; L - lingering pain; ++ - mild pain; -ve - no response; +ve - positive response; 0 - no response

Test/tooth	#21	#22	#23	#25	#26
Cold test	0	L	N	N	N
EPT	-ve	+ve	+ve	+ve	+ve
Percussion	++	N	N	N	N

Statistical analysis

Statistical analyses were conducted using SPSS version 26 (IBM Inc., Armonk, New York). A simple descriptive statistic was used to define the characteristics of the study variables through the form of counts and percentages for the categorical and nominal variables while continuous variables are presented by mean and standard deviations. One-way ANOVA was used to compare specialists as independent variables; management and diagnosis were dependent variables. Lastly, a conventional p-value <0.05 was the criteria to reject the null hypothesis.

## Results

Of 120 sample sizes, 111 participants from different specialties were interviewed, comprising 30 endodontists (27%), 30 oral maxillofacial surgeons (27%), 21 oral medicine specialists and oral pathologists (19%), and 30 general practitioners (27%). Participant ages ranged between 25 to 64 years old, with a mean of 34.6 years old. The majority of the participants were males (65.8%), and 34.2% were females. Moreover, the years of experience after the bachelor's degree ranged from a minimum of one year to 43 years, with a mean of 12.4 years. A detailed description of the demographic data of the interviewees is shown in (Table 2).

**Table 2:** Demographic data *One-way ANOVA test

Variables			Total		Mean±SD	p-value*
			N	%		
			111	100		
General information	Gender	Female	38	34.2	1.66±0.47	0.160
		Male	73	65.8		
	Age	25-34 years	52	46.8	34.68±12.94	0.001
		35-44 years	37	33.4		
		45-54 years	14	12.6		
		55-64 years	8	7.2		
	Level of Degree	Bachelor	47	42.3	0.42±0.49	0.000
		Master	21	18.9	0.19±0.39	0.011
		PhD	30	27	0.27±0.44	0.000
		Fellowship	3	2.7	0.03±0.16	0.656
		Board	10	9	0.09±0.28	0.035
	Specialty	Endodontics	30	27	2.92±1.74	-
		Oral maxillofacial surgeon	30	27		
		Oral medicine/ pathologists	21	18.9		
		General practitioners	30	27		

With regard to the awareness of the American Association of Endodontic diagnostic terminology, 64.9% (n=72) were aware of the diagnostic terminology, with the endodontists representing the highest percentage, followed by the general practitioners. On the other hand, 59% of the oral maxillofacial surgeons were unaware of the AAE terminology (Table 3). 

**Table 3:** Q: Are you familiar with the American Association of Endodontics diagnostic terminology

Specialty		Count (%)	No	Yes	Total
Endodontists		Count	0	30	30
		%	0.0%	41.7%	27.0%
General practitioners		Count	4	26	30
		%	10.3%	36.1%	27.0%
Oral maxillofacial surgeons		Count	23	7	30
		%	59.0%	9.7%	27.0%
Oral Medicine specialist/ oral pathologists		Count	12	9	21
		%	30.8%	12.5%	18.9%
Total		Count	39	72	111
		%	100.0%	100.0%	100.0%

The management of the presented case, as well as the treatment of choice by each specialty, is shown in Table 4. RCT and follow-up were only rejected in 84.7% (n=94) of the participants; the majority were oral maxillofacial surgeons, giving a significant difference between other specialties (p=0.03). Surgical removal and follow-up were chosen by one oral and maxillofacial surgeon. The approval (51.4%, n=57) and rejection (48.7%, n=54) of surgical removal and RCT for selected teeth were comparable, with no significant difference (p=0.44). Rejection of surgical removal and RCT for all teeth associated with the lesion showed (83.8%, n=94), with the endodontists and the general practitioners representing the majority; however, there was no significant difference (p=0.2). The oral maxillofacial surgeons presented the highest percentage, being in favor of surgical removal and RCT for all teeth (p=0.03). Some of the participants (n=29) chose (other) to answer the question. The majority were general practitioners (n=17) who justified their management decision of referring the case to a specialist as it was beyond their limit of practice. Oral maxillofacial surgeons (n=1), oral medicine specialists, and oral pathologists (n=5) would also refer to endodontists, capable of giving a definite diagnosis for the pulpal and periapical condition.

**Table 4:** Q: What is the management of this case? RCT -  root canal treatment *one way ANOVA test

Method	Specialty		Yes	Significance between groups (p-value*)
RCT and follow-up only	Endodontists	Count	9	0.037
		%	52.9%	
	General practitioners	Count	4	
		%	23.5%	
	Oral maxillofacial surgeons	Count	1	
		%	5.9%	
	Oral medicine specialist/ oral pathologists	Count	3	
		%	17.6%	
Total	-	Count	17	-
		%	100%	
Surgical removal and follow-up only	Endodontists	Count	0	0.445
		%	0%	
	General practitioners	Count	0	
		%	0%	
	Oral maxillofacial surgeons	Count	1	
		%	100%	
	Oral medicine specialist/ oral pathologists	Count	0	
		%	0%	
Total	-	Count	1	-
		%	100%	
Surgical removal and RCT for selected teeth	Endodontists	Count	15	0.206
		%	26.3%	
	General practitioners	Count	11	
		%	19.3%	
	Oral maxillofacial surgeons	Count	19	
		%	33.3%	
	Oral medicine specialist/ oral pathologists	Count	12	
		%	21.1%	
Total	-	Count	57	-
		%	100%	
Surgical removal and RCT for all teeth	Endodontists	Count	6	0.030
		%	33.3%	
	General practitioners	Count	1	
		%	5.6%	
	Oral maxillofacial surgeons	Count	9	
		%	50%	
	Oral medicine specialist/ oral pathologists	Count	2	
		%	11.1%	
Total	-	Count	18	-
		%	100%	
Other	Endodontists	Count	6	0.000
		%	20.7%	
	General practitioners	Count	17	
		%	58.6%	
	Oral maxillofacial surgeons	Count	1	
		%	3.4%	
	Oral medicine specialist/ oral pathologists	Count	5	
		%	17.2%	
Total	-	Count	29	-
		%	100%	

In response to whether to perform endodontic treatment for vital symptomless teeth involved within the cyst, 79.3% (n=88) of participants replied with a definite no (p=0.18). Thus, they believe there is no need for excessive treatment to be done if there is a more valid and conservative treatment could be applied. Participants who were selected to perform a combination of surgical excision and endodontic treatment (76.6%) as a method of management preferred to do the RCT before the surgical treatment with a similarity in the proportion between different specialties (p=0.66).

Concerning the prognosis of the studied case, the options were favorable, questionable, unfavorable, and other options. 63.1% responded with a favorable prognosis (p=0.001) as opposed to the other answers (Table 5). 

**Table 5:** Q: What is the overall prognosis of this case? *one way ANOVA test

Outcome	Count (%)	Yes	Significance between groups (p-value*)
Favorable	Count	70	0.001
	%	63.10%	
Questionable	Count	13	0.12
	%	11.70%	
Unfavorable	Count	4	0.325
	%	3.60%	
Other	Count	24	0.006
	%	21.60%	
Total	Count	111	-
	%	100.00%	

## Discussion

Our aim in this study was to investigate the different modalities of management from participants of different specialties regarding vital teeth associated with large cystic lesions. The treatment options included in the case presented to the interviewees were root canal treatment only, surgical removal of the lesion only, a combination of both surgical removal and selective RCT for the involved non-vital teeth, and surgical removal and RCT for all teeth involved in the lesion.

The goal of root canal treatment is to prevent and intercept pulpal/periradicular pathosis and to preserve the natural dentition when affected by pathosis [7]. However, vital pulps associated with pathological lesions have different treatment approaches without any consensus on definite treatment options [8].

Up until the 1960s, endodontists, pathologists, and oral maxillofacial surgeons have all believed that in cases with an apical cyst, a non-surgical treatment by itself is not enough for resolution, and surgery is mandated to reach complete healing [9]. Most endodontists included in this study chose to undergo RCT alone for the diseased causative teeth. The conservative approach by endodontists involved only non-surgical treatment, as several published case reports have proven that this line of management is effective in most cases [10-12]. The removal of bacterial contamination from the root-canal system is believed to be the foremost step in periapical treatment. Therefore, the immunity will take control by the mechanism of apoptosis or programmed cell death leading to gradual shrinking until the complete resolution of the lesion with only RCT [13]. This hypothesis explains why they would prefer this method over the others.

Surgical intervention of the lesion alone was only selected by one oral maxillo-facial surgeon (OMFS) and his justification was that the lesion may be caused by a non-odontogenic cause. It has been previously shown that conservative inoculation of a cystic lesion was not associated with the devitalization of teeth after four years of treatments [14]. On the other hand, around 45-50% of participants have chosen a combination of both methods with endodontic treatment selectively for symptomatic teeth, thus eliminating the source of infection, achieving an almost bacteria-free environment, and cleaning canals with a good apical seal. They believe that a large lesion would not heal with only RCT, and surgical intervention, either by enucleation or marsupialization, is needed for complete eradication of the lesion. In marsupialization, the mechanism works by shrinkage and substantial reduction of the lesion size [15]. For that reason, many practitioners prefer it as it has less collateral morbidity as opposed to enucleation. On the contrary, some will lean toward enucleation as it obtains a well-cleaned environment with proper curettage of all the lining of the lesion that may result in recurrence.

Surgical removal and RCT for all teeth involved in the lesion were selected by 15% of the participants. Their rationale is that the surgical intervention will cause a definite devitalization to all teeth associated with the cyst. Consequently, if the involved teeth are not root canal treated, it may lead to the persistence of periapical infection and defect due to compromised vascularity [16]. RCT before or at the time of surgery will help secure the operated area against afterward intervention that may disturb the healing process either by slowing it down or leaking micro-organism that leads to infection and delayed bone healing [13]. 

Most endodontists and newly graduated general dentists are familiar with the latest endodontic diagnostic terminology of the AAE [17] compared to OMFS and oral medicine/pathologists. This justifies why OMFS would not intervene in endodontic-related decisions without consultation of the endodontist in the diagnosis and treatment planning of teeth associated with large cystic lesions. 

The limitation of this study is that it included a single-case scenario that was barely acceptable to some of the participants. Therefore, it was arduous to add additional cases that would benefit the study by extracting more data and evidence. Setting a standardized protocol for treating cases that contain a large cyst involving vital teeth would be laborious for the reason that each clinical case has its own finding, presentation, and diagnosis, requiring its own individual handling and decision.

A long-term follow-up with retrospective analysis of similar studies is needed to determine the indications of each treatment modality, which will help clinicians regarding the management of vital asymptomatic teeth associated with large cystic lesions. Furthermore, standardized decision-making guidelines and protocols provided by evidence-based practice are needed to deliver the best treatment modality with the least potential complications for the patient's sake. 

## Conclusions

In this study, we found that there is no consensus between endodontists, OMFS, oral medicine specialists and oral pathologists in the management of cases of large cystic lesions involving vital teeth. On the other hand, the AEE diagnostic guidelines are common between only endodontists and general dentists when compared with the other disciplines. Therefore, it needs to be unified and disseminated to facilitate easier referrals. Conclusively, further studies with long-term follow-up study designs are needed to aid in reaching a standardized line of decision-making and management for such cases.

## Appendices

The questionnaire can be found using the following link:

https://docs.google.com/forms/d/e/1FAIpQLSddhZ9FE6qiUDPYW_tO2H7OEA4zjYIGT_kH0RGt4vrtU_lIPg/viewform?usp=share_link
